# Unraveling the nexus of NAD+ metabolism and diabetic kidney disease: insights from murine models and human data

**DOI:** 10.3389/fendo.2024.1384953

**Published:** 2024-05-21

**Authors:** Sisi Yang, Weiyuan Gong, Yujia Wang, Chuanming Hao, Yi Guan

**Affiliations:** ^1^ Department of Nephrology, Huashan Hospital, Fudan University, Shanghai, China; ^2^ Department of Nephrology, Blood Purification Research Center, The First Affiliated Hospital, Fujian Medical University, Fuzhou, China

**Keywords:** diabetes, diabetic kidney disease, nicotinamide adenine dinucleotide, kynurenine 3-monooxygenase, pathophysiology

## Abstract

**Background:**

Nicotinamide adenine dinucleotide (NAD+) is a critical coenzyme involved in kidney disease, yet its regulation in diabetic kidney disease (DKD) remains inadequately understood.

**Objective:**

Therefore, we investigated the changes of NAD+ levels in DKD and the underlying mechanism.

**Methods:**

Alternations of NAD+ levels and its biosynthesis enzymes were detected in kidneys from streptozotocin-induced diabetic mouse model by real-time PCR and immunoblot. The distribution of NAD+ *de novo* synthetic enzymes was explored via immunohistochemical study. NAD+ *de novo* synthetic metabolite was measured by LC-MS. Human data from NephroSeq were analyzed to verify our findings.

**Results:**

The study showed that NAD+ levels were decreased in diabetic kidneys. Both mRNA and protein levels of kynurenine 3-monooxygenase (KMO) in NAD+ *de novo* synthesis pathway were decreased, while NAD+ synthetic enzymes in salvage pathway and NAD+ consuming enzymes remained unchanged. Further analysis of human data suggested KMO, primarily expressed in the proximal tubules shown by our immunohistochemical staining, was consistently downregulated in human diabetic kidneys.

**Conclusion:**

Our study demonstrated KMO of NAD+ *de novo* synthesis pathway was decreased in diabetic kidney and might be responsible for NAD+ reduction in diabetic kidneys, offering valuable insights into complex regulatory mechanisms of NAD+ in DKD.

## 1 Introduction

Diabetic kidney disease (DKD) is a primary cause of end stage of renal disease with great global health burden. By 2045, the diabetic population is projected to reach 700 million worldwide (1), with nearly 40% of these individuals expected to develop DKD (2–4). Despite significant achievements of pharmacotherapy such as sodium-glucose cotransporter 2 inhibitors and nonsteroidal mineralocorticoid antagonists, which have demonstrated improved renal outcome in large-scale clinical trials (5–8), in conjunction with the established use of angiotensin-converting enzyme inhibitor and angiotensin II receptor blockers, the therapeutic options for DKD remain limited. Understanding the pathogenic mechanisms of DKD is of critical to guide clinical interventions.

**Figure 1:**
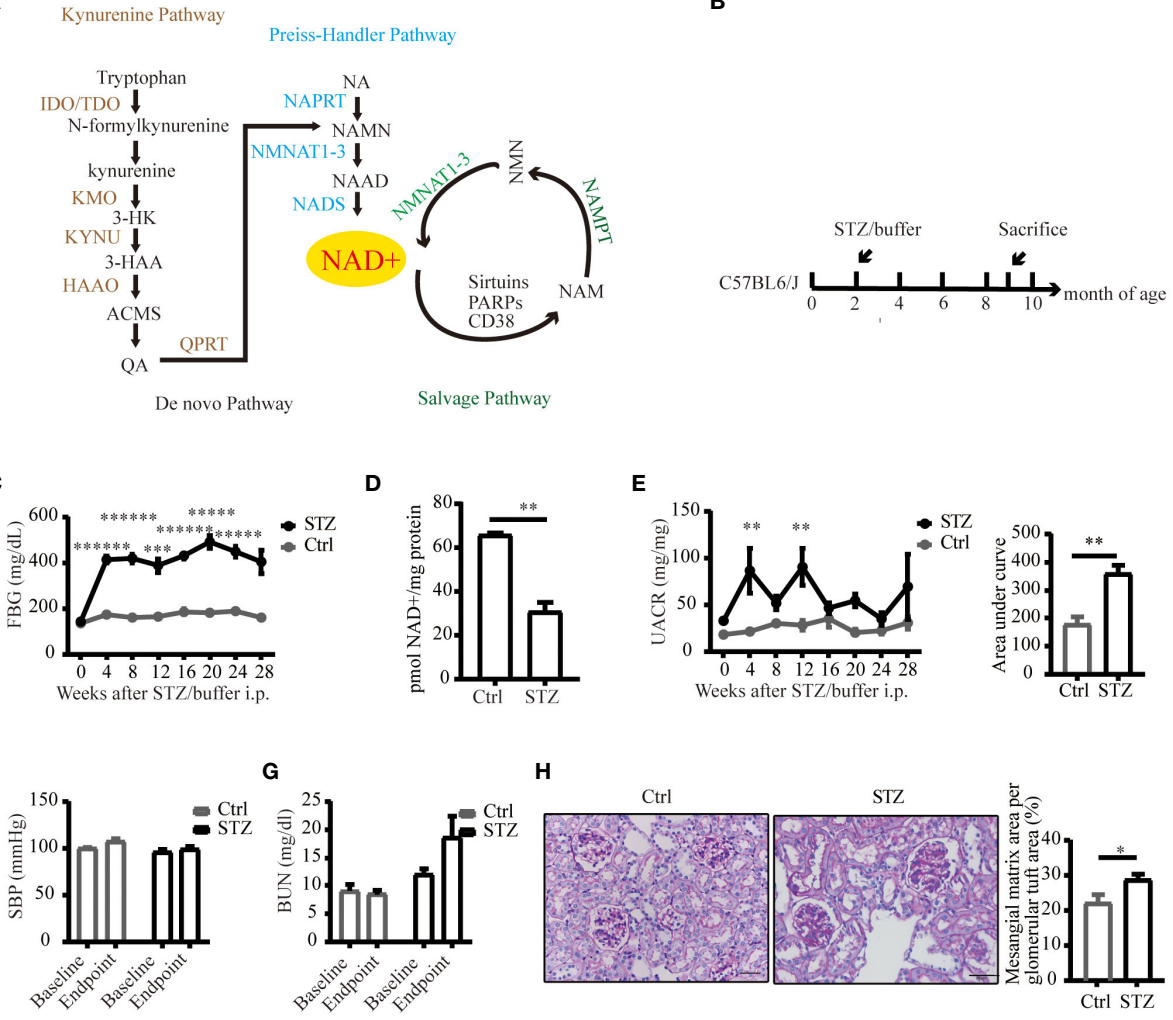
An overview of NAD+ biosynthesis pathways and reduced NAD+ expression in mice kidneys under diabetic conditions. **(A)** Three biosynthetic pathways of NAD+. **(B)** Schematic illustration of the study design for diabetic mouse model establishment. **(C)** FPG levels of C57BL/6J mice following STZ or buffer (n=4–7). **(D)** NAD+ was reduced in diabetic mouse kidneys (n=4 in Ctrl, 6 in STZ). **(E)** Left UACR of spot urine before and every 4 weeks after STZ or buffer administration (n=3–7); Right area under curve of UACR of spot urine after 7 months of STZ or buffer administration (n=4–6). **(F)** Systolic blood pressure before and every 4 weeks after STZ or buffer administration (n=4–7). **(G)** Renal function estimated by BUN before and after STZ or buffer administration (n=4–7). **(H)** Representative images of periodic acid–schiff (PAS)-stained kidney sections (original magnification x400) and semiquantitative analysis of glomerular deposition of PAS positive extracellular matrix after 28 weeks of STZ or buffer administration. The data are shown as means ± SEM. NAD+, nicotinamide adenine dinucleotide; IDO, indoleamine 2, 3-dioxygenase; TDO, tryptophan 2, 3-dioxygenase; KMO, kynurenine 3-monooxygenase; 3-HK, 3-hydroxykynurenine; 3-HAA, 3-hydroxyanthranilic acid; KYNU, kynureninase; HAAO, 3-hydroxyanthranilic acid oxygenase; ACMS, α-amino-β-carboxymuconate-ϵ-semialdehyde; QA, quinolinc acid; QPRT, quinolinate phosphoribosyl transferase; NA, nicotinic acid; NAPRT, nicotinic acid phosphoribosyl transferase; NAMN, nicotinic acid mononucleotide; NMNAT1–3, nicotinamide mononucleotide adenylyltransferase 1–3; NAAD, nicotinic acid adenine dinucleotide; NADS, NAD synthetase; NAM, nicotinamide; NMN, nicotinamide mononucleotide; PARPs, poly (ADP-ribose) polymerases; NAMPT, nicotinamide phosphoribosyl transferase; Ctrl, control; STZ, streptozotocin; FPG, fasting plasma glucose; UACR, urinary albumin-to-creatinine ratio; BUN, blood urea nitrogen; SBP, systolic blood pressure; *P<0.05; **P <0.01; ***P<0.005; *****P< 0.0005; ******P<0.0001.

Nicotinamide adenine dinucleotide (NAD+) is a crucial coenzyme serves in redox reaction across all living cells, and also as a substrate or cofactor for enzymes such as the sirtuins family, poly (ADP-ribose) polymerases (PARPs), and the CD38, intricately participating in cellular metabolism (9, 10). These three classes of NAD+-dependent enzymes, known as NAD+ consuming enzymes, degrade NAD+ to generate a by-product nicotinamide (NAM), which is a biosynthetic precursor of NAD+ and also an inhibitor of their activities. Preclinical studies showed that impaired NAD+ was closely related to kidney diseases (11, 12), boosting NAD+ with precursors has been shown of renal benefits (12–16), suggesting the potential applicability of NAD+-replacement therapy from rodent models to renal patients. To maintain cellular levels, NAD+ can be synthesized in *de novo* and salvage pathway from several distinct dietary precursors (17). In salvage pathway, NAD+ production from all three forms of vitamin B3 including NAM, nicotinic acid and nicotinamide riboside via two-step process. The precursors are converted into an intermediate called nicotinamide mononucleotide (NMN) through nicotinamide phosphoribosyl transferase (NAMPT). Then NMN is converted into NAD+ via nicotinamide mononucleotide adenylyl transferase (NMNAT). *De novo* pathway, also known as the kynurenine pathway, consists of eight steps, and generate NAD+ from dietary tryptophan (TRP) (**Figure 1A**). Although salvage pathway is responsible for most NAD+ production (18, 19), supplementation with TRP has long been recognized as a treatment for vitamin B3 deficiency, suggesting an important role for the *de novo* pathway in NAD+ homeostasis (20). Besides, manipulation of the *de novo* pathway has been shown to change NAD+ levels and resistance to acute kidney injury (AKI) (13, 21–24). Loss-of-function of enzymes in the *de novo* pathway have been reported to result in NAD+ deficiency during embryogenesis and led to congenital renal defects (25), suggesting NAD+ *de novo* biosynthesis pathway as an important factor in renal health. However, the dynamics of NAD+ fluxes in DKD remains unestablished.

In this study, we investigated the alterations in NAD+ levels and the expression of its synthetic and consuming enzymes in diabetic mouse and human kidneys. Additionally, we identified kynurenine 3-monooxygenase (KMO), an enzyme that converts kynurenine (KYN) to 3-hydroxykynurenine (3-HK), as a candidate enzyme in the *de novo* NAD+ synthesis pathway responsible for NAD+ deficiency under diabetic condition. This study aims to enhance our understanding of the intricate molecular mechanisms associated with NAD+ regulation in the context of DKD.

## 2 Materials and methods

### 2.1 Animal studies

Male C57BL/6J mice were purchased from Shanghai Model Organisms Center, Inc. (Shanghai, China) and housed at Fudan University Medical Animal Center with a 12-hour light/dark cycle and provided with standard chow. All animal studies were approved by the Institutional Animal Care and Use Committee of Fudan University. 8-week-old male mice with a C57BL/6J background were intraperitoneally injected with streptozotocin (STZ) (Sigma-Aldrich, 50mg/kg, diluted in citrate buffer, pH = 4.5) for five consecutive days to establish STZ induced diabetic model until 28 weeks after STZ administration when they were sacrificed. Littermates were conducted with the same volume of buffer as control. Fasting plasma glucose (FPG) levels were measured via tail-vein blood using an ACCU-CHEK PERFORMA II glucometer after a 7-hour fast. Mice exhibiting FPG ≥ 200 mg/dL 1week post-STZ injection were considered diabetic. Spot urine samples were used to quantify albumin and creatinine with a commercial ELISA kit (Exocell, Inc. USA), following the manufacturer’s protocol. The urinary albumin to creatinine ratio (UACR) was determined by albumin level and creatinine level from the same samples. Blood urea nitrogen (BUN) was measured using the UREA KIT (liquid; UV-GLDH method; Shanghai Kehua Bioengineering Co., Ltd.). Kidney samples were collected when mice were sacrificed. Blood pressure was measured with the tail-cuff method using a noninvasive automatic blood pressure analyzer (BP-2000 Blood Pressure Analysis System, Visitech Systems, USA) according to the manufacturer’s instructions.

### 2.2 NAD+ measurement

Kidney tissue was extracted and then measured using an NADH/NAD quantification kit (Biovision, USA) according to the manufacturer’s instructions. In brief, ~ 20 mg kidney was washed with PBS, pelleted, and extracted with 400 μL NADH/NAD extraction buffer. 50 μL of extracted samples were used for total NADt detection. Samples were decomposed at 60°C for 30 minutes, cooled, and finally 50 μL transferred to a 96-well plate to measure NADH. A standard curve was prepared with 0, 20, 40, 60, 80, and 100 pmol/well of standard NADH, with the final volume adjusted to 50 μL with NADH/NAD extraction buffer. NAD Cycling Mix composed of NAD cycling buffer and NAD cycling enzyme mix was added to each well at room temperature for 5 minutes to convert NAD to NADH. NADH developer was then added, and the plate was cycled at room temperature for 1–4 hours. OD 450 nm readings were taken, and reactions were stopped with a stop solution. The NADt amount in a sample well was calculated with the equation:


$$(\frac{\left(ODsample\;\left(corrected\right)\right)}{\left(ODsample\;+\;NADH\;Std\left(corrected\right)\right)-ODsample\;\left(corrected\right)\;})*\text{NADH Spike }\left(\text{pmol}\right)$$

### 2.3 Histological analysis of the kidney

Kidneys were fixed in a 4% paraformaldehyde solution, embedded in paraffin, cut into sections with a thickness of 3 μm and then subjected to periodic acid–schiff (PAS) staining. In brief, kidney sections were dewaxed in water, oxidized in 1% periodic acid solution (BaSO, China) for 10 minutes, placed in Schiff’s reagent (BaSO, China) for 20 minutes and then counterstained in Mayer’s hematoxylin for 10 seconds (BaSO, China). The area of mesangium and glomeruli was calculated by pixel counts on a minimum of 10 randomly selected glomeruli per kidney section by ImagePro Plus software in a single-blind fashion.

### 2.4 Western blot

Total protein was extracted from the frozen quartered kidney tissue using RIPA lysis buffer (Beyotime, China) with PMSF (Sigma-Aldrich, USA), protease inhibitor cocktail (Roche, USA) and phosphatase inhibitor cocktail (Roche, USA). Protein concentrations were determined using BCA protein assay kit (Beyotime, China). Equal amount of protein (~20 μg per lane) was loaded in a 7.5% or 10% SDS-PAGE mini-gel and transferred to a PVDF membrane (Millipore, USA). The membrane was blocked with 5% BSA in TBST buffer (100 mM TBS, pH 7.5, 0.1% Tween-20) or 5% nonfat dry milk dissolved in TBST buffer and then incubated in primary antibody overnight at 4°C. The primary antibodies included: anti-SIRT1 antibody (Abcam, 1:1000), anti-CD38 antibody (Proteintech, 1:1000), anti-PARP1 antibody (Proteintech, 1:1000), anti-KMO antibody (Proteintech, 1:1000), anti-QPRT antibody (Proteintech, 1:1000), anti-NAMPT antibody (Millipore, 1:5000), anti-GAPDH antibody (Proteintech, 1:8000). Membranes were then incubated with appropriate secondary antibodies for one hour at room temperature and subjected to chemiluminescence detection using ECL Reagent (Millipore,USA).

### 2.5 RNA isolation and quantitative real-time PCR

The isolation of RNA and real-time PCR were performed as previously described (26). In brief, frozen quartered kidney tissue were processed with TRIzol (Thermo Fisher, USA), chloroform, isopropanol, 70% ethanol, and finally RNA suspension in RNase-free water. PrimeScript™ RT reagent Kit (Takara, Japan) was used to perform cDNA synthesis according to the manufacturer’s protocol. Levels of mRNA were determined by real time qPCR using SYBR Premix Ex Taq Kit (Takara, Japan). The expression levels of the target genes were normalized to GAPDH using the 2^−ΔΔCt^ method.

The primer sets used are shown in **Table 1**.

**Table 1:** Primer sets used in the study.

Name		5’-3’
KMO	Forward	ATGGCATCGTCTGATACTCAGG
	Reverse	CCCTAGCTTCGTACACATCAACT
QPRT	Forward	CCGGGCCTCAATTTTGCATC
	Reverse	GGTGTTAAGAGCCACCCGTT
HAAO	Forward	GAACGCCGTGTGAGAGTGAA
	Reverse	CCAACGAACATGATTTTGAGCTG
KYNU	Forward	GTCAAGCCTGCGTTAGTGG
	Reverse	GGAGGGTTTGAAATTCGGAATCC
NAMPT	Forward	AATGTCTCCTTCGGTTCTGGT
	Reverse	GCAACTGGGTCCTTAAACACA
NMNAT1	Forward	TGGCTCTTTTAACCCCATCAC
	Reverse	TCTTCTTGTACGCATCACCGA
NMNAT3	Forward	ATTGACGGGTGAGATGATGCC
	Reverse	ACTGGATGGGGTGGGAAT
GAPDH	Forward	ACGGCCGCATCTTCTTGTGCA
	Reverse	TGCCACTGCAAATGGCAGCCC

KMO, kynurenine 3-monooxygenase; QPRT, quinolinate phosphoribosyl transferase; HAAO, 3-hydroxyanthranilic acid oxygenase; KYNU, kynureninase; NAMPT, nicotinamide phosphoribosyl transferase; NMNAT1, nicotinamide mononucleotide adenylyltransferase 1; NMNAT3, nicotinamide mononucleotide adenylyltransferase 3; GAPDH, glyceraldehyde-3-phosphate dehydrogenase.

### 2.6 Immunofluorescence and immunohistochemistry analyses

Kidneys were fixed in 4% paraformaldehyde solution, embedded in paraffin, and cut into sections with a thickness of 3 μm. Sections were dewaxed in water and placed in an EDTA antigen retrieval buffer (pH 8.0) for thermal repair. They were then incubated in a 3% H2O2 solution for 30 minutes. For blocking, 3% bovine serum albumin in PBS was used, followed by overnight incubation with primary antibodies at 4°C. Next, a secondary antibody (HRP) was added, and the sections were incubated at room temperature for 30 minutes before diaminobenzidine (DAB) staining controlled under a microscope. The positive staining appeared brownish-yellow in the first round. The second round of immunohistochemistry staining followed the same procedure: antigen retrieval, endogenous peroxidase blocking, and BSA blocking. Target primary antibodies were incubated at 4°C overnight followed by secondary antibody incubation at room temperature for 30 minutes. Finally, a red staining solution working solution (Shanghai RecordbioTechnology Co. Ltd, PB: CU: Red dye: AC= 860:40:1:100) was added and controlled under the microscope. Positive staining in the second round appeared red. Immunofluorescence was conducted on paraffin-embedded kidney sections as previously described (27). The primary antibodies used in immunohistochemistry are as follows: anti-KMO antibody (Proteintech, 1:200), anti-QPRT antibody (Proteintech, 1:200), anti-NCC antibody (Abcam, 1:1000), anti-THP antibody (Santa Cruz, 1:200), anti-AQP2 antibody (Santa Cruz, 1:200). Primary antibody used in immunofluorescence are as follows: anti-KMO antibody (Proteintech, 1:200), anti-QPRT antibody (Proteintech, 1:200), anti-LTL antibody (Vector Laboratories, 1:200).

### 2.7 NAD+ metabolites measurements

Human urinary KYN was quantified using LC-MS. The procedure and specifics of LC-MS conditions were detailed as described in reference (24).

### 2.8 NephroSeq data analysis

The NephroSeq database (www.nephroseq.org) was used to examine the mRNA level of KMO gene. The Lindenmeyer Normal Tissue Panel dataset was employed to discern its distribution in renal compartments among healthy kidney transplant donors. Three datasets were available for the analysis of KMO expression levels between healthy kidney transplant donors and DKD patients. These datasets include Woroniecka Diabetes Glom Dataset, Woroniecka Diabetes TubInt Dataset, and Schmid Diabetes TubInt Dataset.

### 2.9 Statistical analysis

All data were presented as the mean ± SEM. Statistical comparisons were performed using unpaired Student’s t test or ANOVA. GraphPad Prism 8.0 software was used for statistical analysis. A value of P< 0.05 indicated a statistically significant difference.

## 3 Results

### 3.1 NAD+ levels were decreased in diabetic mouse kidneys

To delineate alterations in NAD+ levels within diabetic kidneys, we successfully established a diabetic model of type 1 diabetes induced by STZ (**Figures 1B, C**). Compared to control littermates, NAD+ levels were significantly reduced in diabetic mice kidneys 29 weeks post-STZ administration (**Figure 1D**). Renal impairment was evaluated by ACR of spot urine, BUN and histological examination. As depicted in **Figures 1E, F**, diabetic mice showed increased urine ACR, a sensitive and early indicator of DKD, independent of changes in blood pressure. Although the kidney function biomarker, BUN, showed an increasing trend in the diabetic mice (**Figure 1G**), no statistically significant difference was noted. Histological lesions examined by PAS staining showed more mesangial matrix expansion in diabetic group (**Figure 1H**). Our data suggested mild and early renal impairment was induced by STZ in C57 mice, and NAD+ levels was reduced in these diabetic kidneys.

### 3.2 *De novo* NAD+ synthesis was impaired in diabetic kidneys

**Figure 2:**
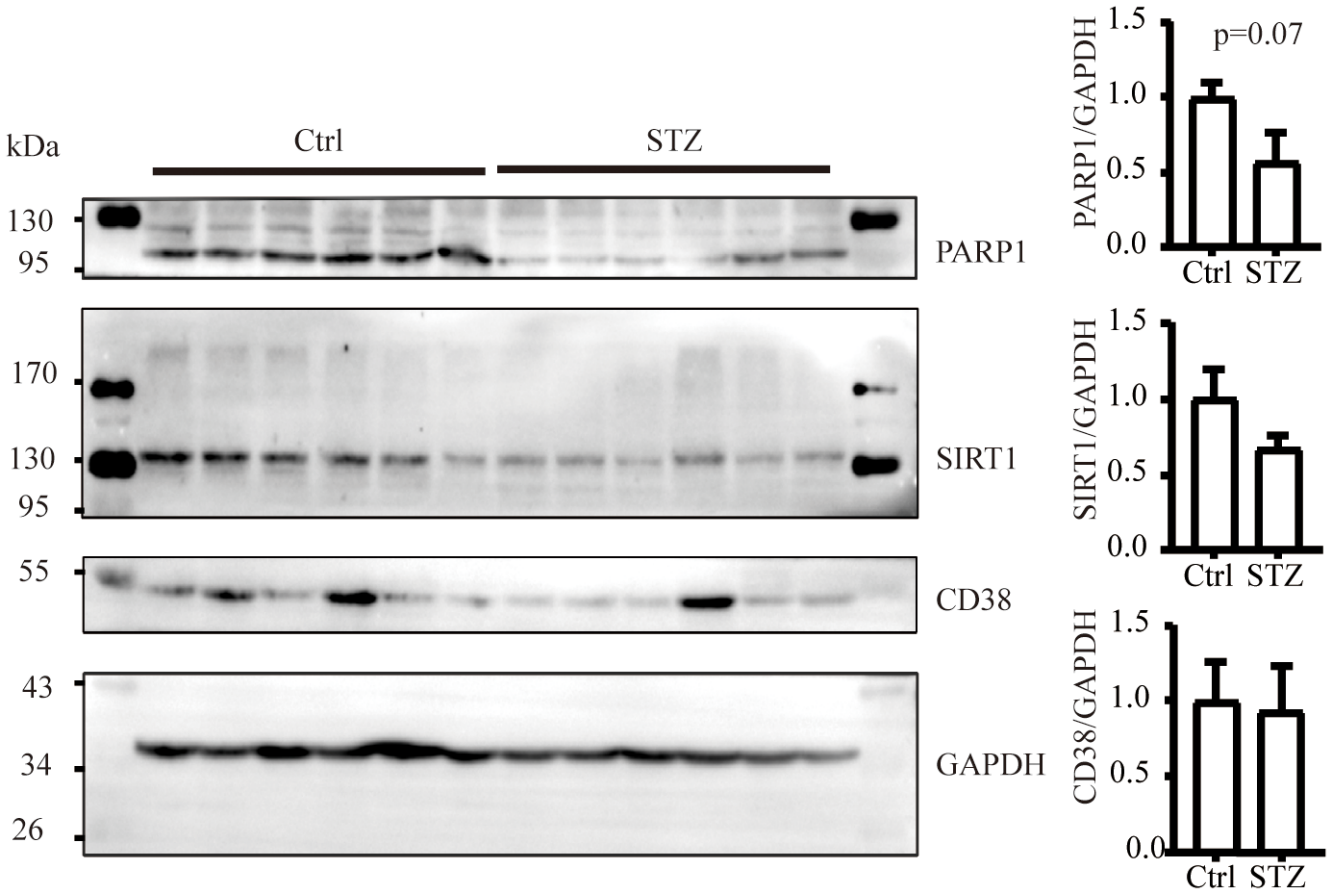
The level of NAD+ consuming enzymes remain unchanged in mice kidneys subjected to diabetic condition (n=6 each group). The data are shown as means ± SEM.

**Figure 3:**
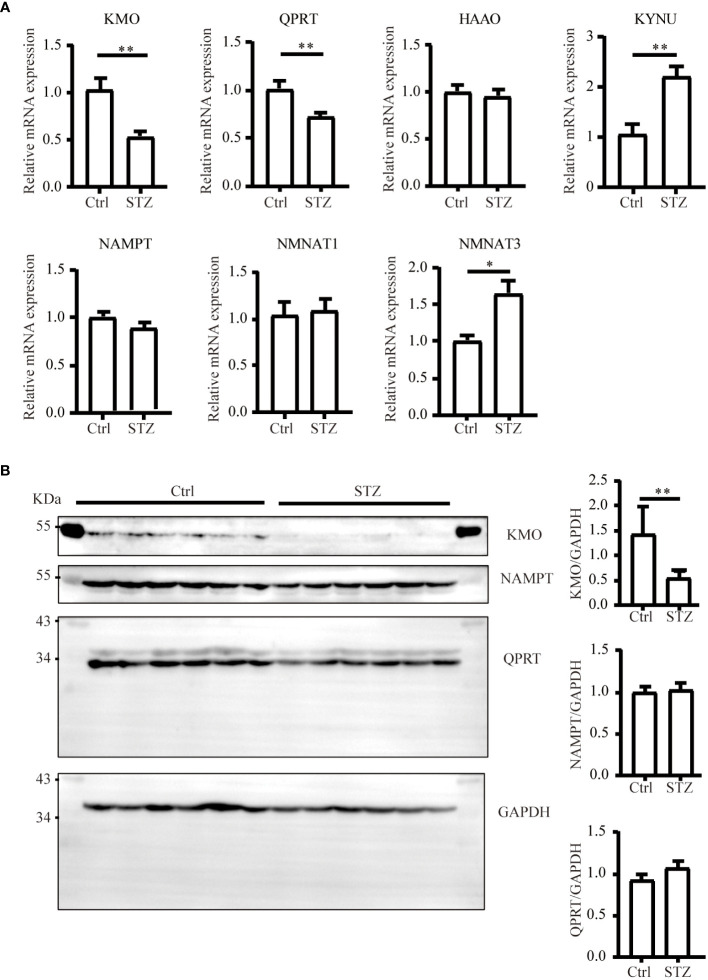
Changes of major enzymes responsible for NAD+ synthesis in mouse kidneys under diabetic condition. **(A)** Real-time quantitative PCR analysis of key enzymes involved in NAD+ synthesis pathways in the kidneys (n=4–6). GAPDH was used as an internal control. **(B)** Representative western blot images and densitometry analysis of KMO, NAMPT, and QPRT protein in mouse kidneys (n=6 each group). The data are shown as means ± SEM. *P<0.05; **P<0.01.

The level of NAD+ is contingent upon a delicate balance between synthesis and consumption (28). In order to explore the underlying causes of the NAD+ decline in diabetic kidneys, we initially examined the levels of NAD+-consuming enzymes. In our study, although a decreasing trend of SIRT1 and PARP1 protein levels was observed in western blot analysis, no significance was calculated. Similarly, there was no change of CD38 levels in diabetic conditions (**Figure 2**). NAD+-dependent deacetylase SIRT1 was reported to be reduced in high energy environment as diabetic kidneys, leading to transcription factor acetylation and kidney lesion (29). SIRT1 has a high Km value for NAD+ compared to other NAD+ consuming enzymes, meaning SIRT1’s activity is highly dependent on NAD+ availability but contributes little to NAD+ consumption (30). Since indifferent expression of NAD+-consuming enzymes could not lead to NAD+ deficiency, then, we assessed the changes of key enzymes in different NAD+ synthesis pathways in diabetic kidneys by mRNA quantification. Compared to the control, the mRNA levels of KMO, a rate-limiting enzyme that transfer KYN into 3-HK, and quinolinate phosphoribosyl transferase (QPRT), an enzyme that catalyze quinolinic acid (QA) into NMN in the *de novo* synthesis pathway, were decreased after STZ administration (**Figure 3A**). In the salvage pathway, no significant decrease of NAMPT nor NMNAT1 were observed in diabetic kidneys, with the exception of upregulation of NMNAT3. These findings suggested a contributory role of *de novo* pathway in NAD+ dynamics when exposed to diabetes. WB analysis further confirmed the reduction of KMO protein in the kidneys of the diabetic group (**Figure 3B**). Taken together, our data implied that the downregulation of KMO in *de novo* pathway might lead to a decrease in NAD+ levels.

### 3.3 KMO was expressed in the proximal tubules

**Figure 4:**
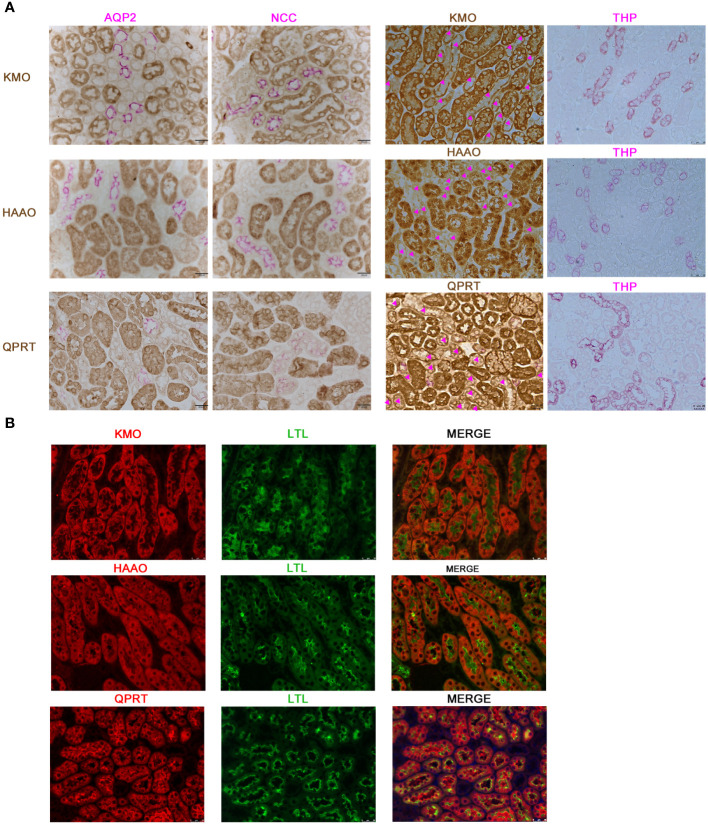
Characterization of KMO, HAAO, and QPRT in mouse kidneys. **(A)** Representative immunohistochemical co-staining of KMO, HAAO, and QPRT (brown) with renal tubular markers (pink) in mouse renal (magnification as 400×). **(B)** Immunofluorescence co-staining (red) of KMO, HAAO, and QPRT with renal tubular markers (green) (magnification as 400×). AQP2, aquaporin 2; NCC, sodium-chloride cotransporter; THP, Tamm-Horsfall protein; LTL, lotus tetragonolobus lectin.

Considering the potential value of KMO in DKD, we further investigated the localization of KMO within the mouse kidney. Immunohistochemical co-staining results revealed that KMO did not colocalize with aquaporin 2 (AQP2), a recognized marker of the collecting duct, nor with sodium-chloride cotransporter (NCC), a marker for the distal convoluted tubule, or with Tamm-Horsfall protein (THP), a marker of thick ascending limb of the renal medulla (**Figure 4A**). KMO was predominantly expressed in the cytoplasm of the proximal tubule, as evidenced by immunofluorescence analysis with Lotus tetragonolobus lectin (LTL) (**Figure 4B**). Moreover, QPRT and 3-hydroxyanthranilic acid oxygenase (HAAO), two other pivotal enzymes with predictive value of AKI in the *de novo* pathway (21, 22, 24), were also present in the same pattern. These observations indicated that the proximal tubule may serve as the primary site for the *de novo* synthesis pathway of NAD+ in the mouse kidneys.

### 3.4 KMO expression in DKD patients

**Figure 5:**
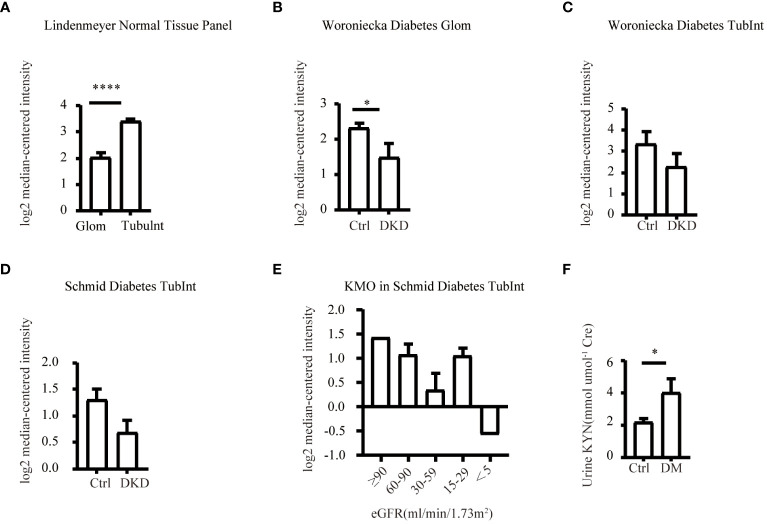
Characterization of KMO in human kidneys. Comparative analysis of KMO and mRNA expression in human kidneys evaluated by microarray method. **(A)** in renal glomeruli (Glom) and tubulointerstitium (TubuInt) from lindenmeyer Nomal Tissue Panel dataset (n=6); **(B)** in renal glomeruli from Woroniecka Diabetes Glom dataset (n=9–13); **(C)** in renal tubulointerstitium from Woroniecka Diabetes TubInt dataset (n=10–12); **(D)** in renal tubulointerstitium from Schmid Diabetes TubInt dataset (n=3–11); **(E)** KMO expression in diabetic nephropathy with eGFR from Schmid Diabetes TubInt dataset (n=1–4). **(F)** Urinary KYN in healthy controls and diabetic patients (n=55 in Ctrl, 16 in DM). DKD, diabetic kidney disease; DM, diabetes mellitus; KYN, kynurenine. The data are shown as means ± SEM. *P<0.05; ****P<0.001.

Our study suggested that the diminished NAD+ levels in diabetic mouse kidneys may be due to a reduction of KMO in the *de novo* synthesis pathway. To explore the distribution of KMO in human kidneys along with its alteration in DKD patients, we utilized the NephroSeq dataset to analyze the transcriptional level of KMO. A total of four datasets, the patient characteristics of which are detailed in **Tables 2**–**5**, were available for analysis. As illustrated in **Figure 5A**, microarray data form the Lindenmeyer Normal Tissue Panel dataset revealed the expression of KMO in both glomeruli and tubulointerstitium, with a more pronounced expression observed in the tubulointerstitium, suggesting the tubulointerstitium as a pivotal site for NAD+ *de novo* synthesis in human kidney align with the mouse data. A significant decrease in KMO was found in DKD patients when compared to healthy living donors in isolated glomeruli (**Figure 5B**). However, in the tubulointerstitium region, although a downward trend in KMO expression was observed across two datasets, the changes were not statistically significant, likely attributed to the limited sample size (**Figures 5C, D**). Analysis of KMO according to eGFR level from Schmid Diabetes TubInt dataset showed a KMO decreased as eGFR fell (**Figure 5E**). Furthermore, an accumulation of urinary KYN, a metabolite upstream of KMO, was observed in diabetic population by LC-MS (**Figure 5F**). These data altogether indicated that renal KMO was decreased in diabetic kidney and played a potential role in pathogenesis of DKD.

**Table 2:** Basic information of Lindenmeyer Normal Tissue Panel.

	Healthy Living Donor (n=6)
Male, n (%)	3 (50)
Age, years	48.56 ± 4.67

**Table 3:** Basic information of Woroniecka Diabetes Glom dataset.

	Healthy Living Donor (n=13)	Diabetic Nephrology (n=9)
Male, n (%)	8 (61.54)	4 (44.44)
Age, years	51.38 ± 3.31	64 ± 4.52
eGFR, ml/min/1.73m^2^	80.91 ± 6.50	31.08 ± 4.46

**Table 4:** Basic information of Woroniecka Diabetes TubInt dataset.

	Healthy Living Donor (n=12)	Diabetic Nephrology (n=10)
Male, n (%)	6 (50)	2 (20)
Age, years	54.08 ± 3.99	63.5 ± 4.95
eGFR, ml/min/1.73m^2^	73 ± 6.09	21.86 ± 3.65

**Table 5:** Basic information of Schmid Diabetes TubInt dataset.

	Healthy Living Donor (n=3)	Diabetic Nephrology (n=11)
Male, n (%)	3 (100)	8 (72.73)
Age, years	39	58.36
eGFR, ml/min/1.73m^2^	87 ± 10.54	48.64 ± 7.97
Hb (%)	No data	7.11 ± 0.37
Proteinuria, g/24h	< 0.2	2.95 ± 0.80

## 4 Discussion

The current study identified a decrease of renal NAD+ level in diabetic mice of early DKD injury phenotype manifested with increased UACR level and mesangial matrix expansion. The downregulation of NAD+ in diabetic kidneys seems primarily attributed to the insufficient biosynthesis through the *de novo* synthesis pathway shown by reduced KMO expression in diabetic kidneys and decreased urinary metabolites of KMO in diabetic patients.

NAD+ functions not only as a universal electron acceptor in glycolysis and the Krebs cycle but also as a substrate for non-redox enzymes that consume NAD+ such as PARPs, sirtuins and CD38 (31, 32). Dysregulated NAD+ levels contribute to the pathogenesis of metabolic disorders, neurodegenerative conditions, and tumorigenesis (9, 33). Our previous study demonstrated that decreased NAD+ levels in aged kidneys contribute to susceptibility to renal injury and replenishing NAD+ by MNN rescued the renal injury in a SIRT1-dependent manner (15). The declined NAD+ levels in DKD, as shown by ours and other (12) in STZ-induced diabetic C57 mice, might lead to disturbed maintenance of mitochondrial function and acetylation of transcriptional factors like PGC-1α by suppressed SIRT activity (34), resulting in renal impairment. Furthermore, NAD+ augmentation has also been reported to attenuate diabetic albuminuria (14, 35), despite renal NAD+ remained unchanged in db/db mice. Short-term NMN supplementation was demonstrated to lead to exerted long-term renal protective effects, and this effect was associated with the increase in NAD+ levels after NMN administration (14). The discrepancy in NAD+ levels might be due to different diabetic animal models and distinct mouse strains. In the kidney, the salvage pathway dominates NAD+ biosynthesis (18, 19). Overexpression of NAMPT, an enzyme of NAD+ salvage pathway, was reported to protect diabetic kidney injury via regulating renal NAD+ level and NAD+-dependent deacetylase SIRT6 (12). In addition to traditional perspectives, recent studies also emphasize the regulatory role of *de novo* synthesis in NAD+ dynamics. Faivre A et al. (36) showed that enzymes involved in the *de novo* NAD+ synthesis pathway were downregulated in kidney allograft patients after transplantation and in ischemia-reperfusion or unilateral ureteral obstruction mice. QPRT deficiency (21, 22) or α-amino-β-carboxymuconate-ϵ-semialdehyde decarboxylase increase (23) induce AKI injury. Additionally, urinary/and plasma metabolites such as QA/3-hydroxyanthranilic acid ratio (24) and QA/TRP (21, 22) are proposed as potential predictive biomarkers for AKI/and AKI progression to CKD. However, these studies predominantly focus on AKI, leaving the dynamics of NAD+ level alterations in CKD, particularly DKD, and the intricate mechanisms underlying its complex downregulation unclear. Our data firstly showed the expression of NAD+ biosynthetic enzymes expression in diabetic murine kidneys. Our study revealed that the *de novo* pathway was impaired in DKD while the enzymes of the salvage pathway remained. Specially, QPRT and KMO were found reduced in transcription level, and the latter was further confirmed decreased in protein level by immunoblot analysis.

Given that KMO deficiency was reported to cause proteinuria in zebrafish and mice (37), we speculated that KMO is involved in the pathogenic mechanisms of DKD and explored the distribution of KMO in murine kidneys. In our study, KMO was predominantly expressed in proximal tubules, with the other two key enzymes of *de novo* NAD+ synthesis pathway, QPRT and HAAO, implying proximal tubules as active sites for *de novo* NAD+ synthesis to fulfill its high demand for energy consumption, consistent to present human data and our previous human study (24). KMO has also been reported in mice glomeruli, particular in podocyte (37, 38). However, we did not observe podocyte KMO expression in our study, possibly due to the overwhelmingly strong expression of tubular KMO in complete cortex sections.

A meaningful study conducted by Hasegawa et al. demonstrated SIRT1 and NAD+ metabolism alterations in proximal tubules (PTs) occur at a very early stage in diabetes and crosstalk between PTs and podocytes mediated by NAD+/SIRT1 initiated diabetic kidney lesions (35). Our data supplemented their study with evidence that decreased tubular KMO might be responsible for disruption of glomerular renal NAD+ homeostasis in early phase of DKD. This was further supported by the work of Yougang Zhai et al., wherein the overexpression of KMO in primary cultured human proximal tubular cells exhibited otherwise undetectable downstream intermediate metabolites such as 3-HK, QA and consequently restored the NAD+ levels originating form *de novo* pathway (19).

The limitations of this study include the absence of gain and loss of KMO function studies to validate KMO’s role in diabetic condition. And, a more comprehensive metabolic profiling of substrate and enzyme activity investigations of the *de novo* pathway need to be addressed in subsequent experiments to refine our understanding.

In conclusion, our study demonstrated that NAD+ was decreased in DKD. Reduced KMO in NAD+ *de novo* synthesis pathway under diabetic condition contributed to the NAD+ deficiency, suggesting KMO as a potential therapeutic target for DKD. These findings contribute to our evolving understanding of DKD pathophysiology and suggest potential avenues for targeted interventions to mitigate renal injury and improve patient outcomes.

## Data availability statement

The original contributions presented in the study are included in the article/supplementary material. Further inquiries can be directed to the corresponding author.

## Ethics statement

The studies involving humans were approved by Huashan Hospital, Fudan University. The studies were conducted in accordance with the local legislation and institutional requirements. The participants provided their written informed consent to participate in this study. The animal study was approved by Institutional Animal Care and Use Committee of Fudan University. The study was conducted in accordance with the local legislation and institutional requirements.

## Author contributions

SY: Formal analysis, Methodology, Visualization, Writing – original draft. WG: Formal analysis, Methodology, Writing – original draft. YW: Formal analysis, Writing – original draft, Validation. CH: Conceptualization, Funding acquisition, Project administration, Resources, Writing – review & editing, Supervision. YG: Conceptualization, Funding acquisition, Project administration, Supervision, Visualization, Writing – review & editing.
